# Frailty in a racially and socioeconomically diverse sample of middle-aged Americans in Baltimore

**DOI:** 10.1371/journal.pone.0195637

**Published:** 2018-04-10

**Authors:** Felicia R. Griffin, Nicolle A. Mode, Ngozi Ejiogu, Alan B. Zonderman, Michele K. Evans

**Affiliations:** 1 Department of Statistics, Florida State University, Tallahassee, Florida, United States of America; 2 Laboratory of Epidemiology and Population Sciences, National Institute on Aging, National Institutes of Health, Baltimore, Maryland, United States of America; Cardiff University, UNITED KINGDOM

## Abstract

Frailty is a risk factor for disability and mortality, and is more prevalent among African American (AA) elderly than whites. We examine frailty in middle-aged racially and economically diverse adults, and investigate how race, poverty and frailty are associated with mortality. Data were from 2541 participants in the Healthy Aging in Neighborhoods of Diversity across the Life Span study in Baltimore, Maryland; 35–64 years old at initial assessment (56% women; 58% AA). Frailty was assessed using a modified FRAIL scale of fatigue, resistance, ambulation, illness and weight loss, and compared with difficulties in physical functioning and daily activities. Frailty prevalence was calculated across race and age groups, and associations with survival were assessed by Cox Regression. 278 participants were frail (11%); 924 pre-frail (36%); 1339 not frail (53%). For those aged 45–54, a higher proportion of whites (13%) than AAs (8%) were frail; while the proportions were similar for those 55–64 (14%,16%). Frailty was associated with overall survival with an average follow-up of 6.6 years, independent of race, sex and poverty status (HR = 2.30; 95%CI 1.67–3.18). In this sample of economically and racially diverse older adults, the known association of frailty prevalence and age differed across race with whites having higher prevalence at younger ages. Frailty was associated with survival beyond the risk factors of race and poverty status in this middle-aged group. Early recognition of frailty at these younger ages may provide an effective method for preventing or delaying disabilities.

## Introduction

Frailty is generally regarded as a clinical syndrome in the elderly associated with increased risk for disability, hospitalization, and mortality [1]. Although a medical condition, frailty is not synonymous with comorbidities or merely the product of age-associated functional declines [2]. Operational definitions of frailty are numerous and yield differing estimates of frailty prevalence [3]. The International Academy of Nutrition and Aging (IANA), in an effort to build a consensus screening tool, proposed a FRAIL scale with five domains: fatigue, resistance, ambulation, co-morbid illnesses and weight loss [4, 5]. This scale was proposed as a brief screening tool to identify persons at risk for frailty [5] and has shown predictive value for future disabilities and mortality [6–8]. Similar to the frail phenotype proposed by Fried [9] and colleagues, the FRAIL scale identifies an intermediate, or pre-frail, category. Pre-frailty describes prodromal frailty, which with time is likely to meet the criteria. Pre-frailty is also a risk factor for morbidity, including cardiovascular disease [9], and mortality [10, 11].

The FRAIL scale is a screening tool easily applied in small clinical settings, making it appropriate for minority and low-income populations which may be at higher risk for frailty [1, 12]. Its application requires no specialized equipment or population reference values, as required for grip strength and walking speed [9], and it has been successfully used by non-healthcare professionals in community screening [13]. The FRAIL scale has been validated in an African American population, and was associated with mortality after 9 years [6, 7], although survival was not examined. The scale has not been assessed across economically diverse and comparable African American and white groups to examine potential disparities based on race or poverty. We use the Healthy Aging in Neighborhoods of Diversity across the Life Span (HANDLS) study to examine the association of race and poverty with FRAIL scale scores and how sex, race, and poverty may modify the relationship of frailty and survival.

## Methods

### Sample

HANDLS is a prospective longitudinal study of age-associated health disparities in an urban-dwelling, socioeconomically diverse cohort of African American and white adults living in Baltimore City, Maryland [14]. Participants were recruited by sampling age (seven 5-year age-bands, 30–64), sex, race (African American or white), and poverty status indexed by 125% of the Federal Poverty level. Enrollment from 13 neighborhoods and initial data collection began in August 2004 and ended in November 2008 with 3,720 participants. Approval for data collection was obtained from MedStar Institutional Review Board and the National Institutes of Health, National Institute of Environmental Health Sciences Review Board. All participants provided written informed consent.

This study included participants who were initially 35–64 years old (n = 3325), of whom 2541 had sufficient data to determine frailty and were used for analysis (mean age 50.2 years; 56% women; 58% African American; 40% below 125% of the federal poverty limit). People who lacked sufficient data were more likely to be African American (64%, p = 0.003), male (51%, p = 0.001), and slightly younger (49.4 years, p = 0.010), but did not differ in poverty status (p = 0.121) or self-rated health (p = 0.419).

### Baseline measures

Age, biologic sex, self-reported race, and poverty status based on household income were collected as part of enrollment in HANDLS. Educational attainment was based on self-reported years of education and dichotomized into those who graduated high-school or earned an equivalency and those with less than 12 years of education. Body mass index (BMI) was assessed as weight/height^2^ (kg/m^2^) where weight and height were measured using a standard protocol by medical staff. Body weight was measured without shoes and coats using a calibrated Health O Meter digital scale (Pelstar, LLC, Alsip, IL). Standing height was measured directly on a calibrated scale with a ruler set parallel to the top of the participant’s head. BMI was classified into five groups: underweight (BMI<18.5), normal (18.5≤BMI<25), overweight (25≤BMI<30), obesity class 1 (30≤BMI<35), and obesity class 2 (BMI≥35).

### Mortality data

Mortality data were derived from the National Death Index through December 31, 2013. Minimal loss of mortality information was expected since all but two participants (2539) provided a social security number. Follow-up time was censored based on last known date alive, with 84% of the participants having at least one additional visit; 42 people had no additional contact and were not included in the mortality analyses. There were 230 deaths with an average follow-up time of 6.6 years (median 6.6 years, maximum 9.4 years), and a total of 16,468 person-years (PY).

### Frailty

The FRAIL scale consists of five domains [4]: fatigue, resistance (ability to climb stairs), ambulation (ability to walk a certain distance), number of illnesses, and loss of weight. We followed the measurements of Morley et al. [6] except for loss of weight which was adapted as per Theou et al. [3]. Fatigue was measured from responses to item 20 of the Center for Epidemiologic Studies Depression scale (CES-D) [15]. Over the past week did you feel you could not get going?) and considered it present when participants responded occasionally (3–4 days a week) or mostly (5–7 days a week). Resistance was assessed by whether participants reported any difficulty walking up 10 stairs. Ambulation was assessed by whether participants reported difficulty walking a quarter of a mile. Illness was assessed as positive reports of five or more conditions out of 11 during a structured medical history (Has a doctor ever told you that you have hypertension, diabetes, cancer, chronic lung disease, heart attack, congestive heart failure, angina, asthma, arthritis, stroke, and kidney disease). We assessed loss of weight from responses to item two of the CES-D (Over the past week did you not feel like eating or have a poor appetite?). Weight loss was considered present when participants responded occasionally (3–4 days a week) or mostly (5–7 days a week). *Frail scores* are the number of components present and range from 0 (all components absent) to 5 (all components present). Frail score was categorized into three *frailty groups*: frail (frail score 3–5), pre-frail (1–2), or not frail (0). *Frailty status* was dichotomized as frail (score 3–5) versus not (0–2). Participants were required to have data on at least three of the five components to be included in the sample, similar to the criteria used for the frailty phenotype [9].

To ensure comparability with other versions of the FRAIL scale, we also examined the concurrent validity of the FRAIL scale in HANDLS. We tested the association of the frailty groups with measures related to the frailty clinical syndrome including self-reported physical functioning; functional status assessed by instrumental activities of daily living (IADL); and grip strength, a physical performance measure. Health status measures included self-reported health and polypharmacy. Self-rated health was dichotomized as poor or fair versus good, very good, or excellent responses to the overall health item from the SF-12 [16]. Participants reported all current prescription medications, presenting actual medications when possible, to the medical staff during their visit. Polypharmacy was considered the current (within two-weeks) use of six or more medications [17].

Physical functioning was assessed through self-reported difficulties in performing seven activities: standing from a chair; carrying 10 pounds; stooping, crouching, or kneeling; pulling or pushing objects; doing heavy housework; using fingers; and raising hands. Each activity was scored as 0 (no difficulty) or 1 (have difficulty). Physical function difficulty was a sum score greater than 0, or difficulty in any area. The IADL is composed of seven self-reported assessments describing the need for assistance in doing chores around the house, preparing own meals, and managing money, ranging from 1 (can do the task without help) to 3 (unable to do the task). The scores are summed with totals of 8 or greater indicating some level of disability.

Trained technicians assessed dominant-hand grip strength using a Jamar Hydraulic hand dynamometer (Model No. 5030J1Sammons Preston Rolyan, Bolingbrook, IL). Grip strength was measured twice with a 15 to 20 second rest between trials; the mean value was used for analysis. The test was not performed if the participant reported surgery within the past three months or if they had pain or arthritis that would impede their successful completion of the task.

### Data analyses

The level and pattern of frailty was examined in this middle-aged, racially and economically diverse sample of adults. Demographic variables and health status measures were tested for differences across the three frailty groups by chi-squared goodness of fit tests for categorical variables and analysis of variance (ANOVA) for continuous variables. To examine how frailty was associated with race and age in the HANDLS sample, the prevalence of frailty status was assessed across the race and age groups by chi-squared goodness of fit tests. The relationship of frailty status with all the variables of interest (sex, race, age, poverty status, BMI, educational attainment) was modeled using multivariable logistic regression. Forward and backward variable selection was performed including two- and three-way interactions to build the final model. Odds ratios (OR) and 95% confidence intervals (CI) are presented.

The concurrent validity of the FRAIL scale used in this study was tested through association with physical functioning difficulties, IADL difficulties and grip strength. Differences for each measure across the three frailty groups were tested using chi-squared goodness of fit (physical functioning, IADL) and ANOVA (grip strength). In addition, we assessed the association of these variables with frailty status after accounting for sex, race, age, poverty status, BMI and educational attainment. Logistic regression was used for difficulties in physical functioning and IADLs. Associations with grip strength were tested using ANOVA and calculated effect sizes (η^2^) are presented.

The relationship of frailty and survival was examined alone and in the context of other variables of interest. Survival across the three frailty groups both overall and stratified by three 10-year age groups was assessed by Score tests. Multivariable Cox proportional hazards models were used to examine frailty status with the variables of interest: sex, poverty status, race, BMI, and educational attainment. Time was measured by exact age at entrance and exit of the study which was either date of death, date of last contact or 31 Dec 2013. Forward and backward variable selection was performed using likelihood ratio tests to identify significant interactions (two-, three- and four-way) and build the final model. Hazard ratios (HR) are presented for significant variables and interactions. Statistics are provided with confidence intervals and a two-sided p < 0.05 significance level was used for all analyses. Data management and analyses were performed in R software version 3.3 [18].

## Results

Frail participants comprised 11% of the sample (278 people), while 36% were pre-frail (924). Overall, the proportion of frail, pre-frail and not frail participants differed by sex, poverty status, educational attainment, and self-rated health, but not race (Table 1). The mean age and BMI of participants also differed across the three frailty groups. Frail individuals were more likely older, female, below poverty status, with lower educational attainment, lower in self-rated health, taking more than six prescription medications, and having a greater BMI.

**Table 1:** Distribution of demographic variables and health measures by frailty status.

Measure	All (n = 2541)	Not frail (n = 1339)	Pre-frail (n = 924)	Frail (n = 278)	p
Age, mean	50.2	49.5	50.6	52.7	<0.001
Men, %	44	50	39	29	<0.001
African American, %	58	58	59	54	0.319
Below poverty, %	40	32	48	58	<0.001
BMI, mean	30.0	28.9	30.7	33.3	<0.001
HS graduate^1^, %	67	75	60	53	<0.001
Poor or fair health^2^, %	28	13	38	73	<0.001
Polypharmacy^3^, %	14	6	16	41	<0.001

^1^High-school graduate or obtained GED, missing = 67

^2^Poor or fair (versus good, very good, or excellent) self-rated health from SF-12[16], missing = 1

^3^Reported taking six or more prescription medications at time of visit

Overall, the prevalence of frailty status increased with age from 7.2% for 35–44 year olds, to 10.0% for 45–55 year olds, and to 15.4% in those 55–64 years old. The overall prevalence of frailty was similar across African Americans (10.2%) and whites (12.0%), although there were age-related differences by race for those aged 45–54 years (Table 2). Examining the oldest participants compared to the rest, in African Americans the prevalence of frailty was significantly lower before age 55 (7.3%) compared to those 55–64 years old (16.4%; p<0.001). However, in whites, the frailty prevalence was not significantly different for those 35–54 years old (11%), and those 55–64 years old (14.2%). Examining the association of frailty status with the three age groups, sex, race, poverty status, BMI and educational attainment after backward elimination of non-significant interaction terms resulted in a model where the break at age 55 was significant in an interaction with race. The logistic model found female sex (OR = 1.63; 95%CI 1.23–2.18), below poverty status (OR = 2.34; 95%CI 1.79–3.08), BMI (OR = 1.05; 95%CI 1.03–1.06) and age group (<55, 55+) by race (p = 0.022) significantly associated with frailty status. For those under age 55 at enrollment, white participants had an increased odds of being frail compared with African American participants (OR = 1.84; 95%CI 1.30–2.60), while there was not a significant difference by race for those age 55 or older (OR = 0.98: 95%CI 0.65–1.48). Thus, the observed differential prevalence of frailty status by age group and race persisted when sex, BMI, poverty status and educational attainment were included in the model, specifically for those under age 55.

**Table 2:** Frailty prevalence by race and age group.

Age group	African American (N = 1466)	White (N = 1075)	p-value^1^
35-44years, %^2^	6.8	7.6	0.808
45-54years, %	7.7	13.4	0.004
55-64years, %	16.4	14.2	0.446

^1^Chi-squared test

^2^Percent frail within group

Since the weight loss assessment for the FRAIL scale was different than that used by Morley et al. [6], we examined the concurrent validity of the FRAIL measure within HANDLS. Measures of functional status differed across the three frailty groups (Table 3). Levels of frailty were associated with increased odds of having a difficulty in physical functioning (difficulties in carrying, pulling or pushing objects, crouching or kneeling, and using hands and fingers) compared to not frail, after accounting for sex, race, poverty status, BMI, educational attainment and age group (Frail OR = 35.88, 95%CI 18.51–80.61; Pre-frail OR = 3.72, 95%CI 3.04–4.56). Levels of frailty were also associated with difficulties in daily functioning as measured by the IADL (Frail OR = 18.52, 95%CI 12.81–27.10; Pre-frail OR = 5.53, 95%CI 4.05–7.67) and grip strength (η^2^ = 0.01, 95%CI 0.006–0.028) with those in the frail group having the weakest grip, after accounting for sex, race, poverty status, BMI, educational attainment and age group. The proportion of missing observations for grip strength was not uniform across frailty groups, with the highest proportion of missing in the frail group (41%) and fewer missing in pre-frail (29%) and not frail (26%) groups (χ^2^ = 27.2, p<0.001). Variation in grip strength was primarily accounted for by the sex of the participant (η^2^ = 0.41, 95%CI 0.404–0.465), although those with missing data were more likely to be below poverty and African American.

**Table 3:** Concurrent functional measures by frailty group.

Measure	Not frail (n = 1339)	Pre-frail (n = 924)	Frail (n = 278)	p-value^1^
Physical function difficulty^2^, %	38	70	97	<0.001
IADL Difficulty^3^, %	4	24	55	<0.001
Grip strength^4^, mean	35.9	32.3	29.7	<0.001

^1^ Chi-squared goodness of fit test

^2^Physical functioning assessed across seven possible difficulties: standing from chair; carrying 10 pounds; stooping, crouching, or kneeling; pulling or pushing objects; doing heavy housework; using fingers; raising hands. Indicator of any difficulty. Missing = 244.

^3^IADL: Instrumental Activities of Daily Living difficulty indicates self-reported problem in performing at least one of the seven items. Missing = 65.

^4^Missing = 722.

**Fig 1:**
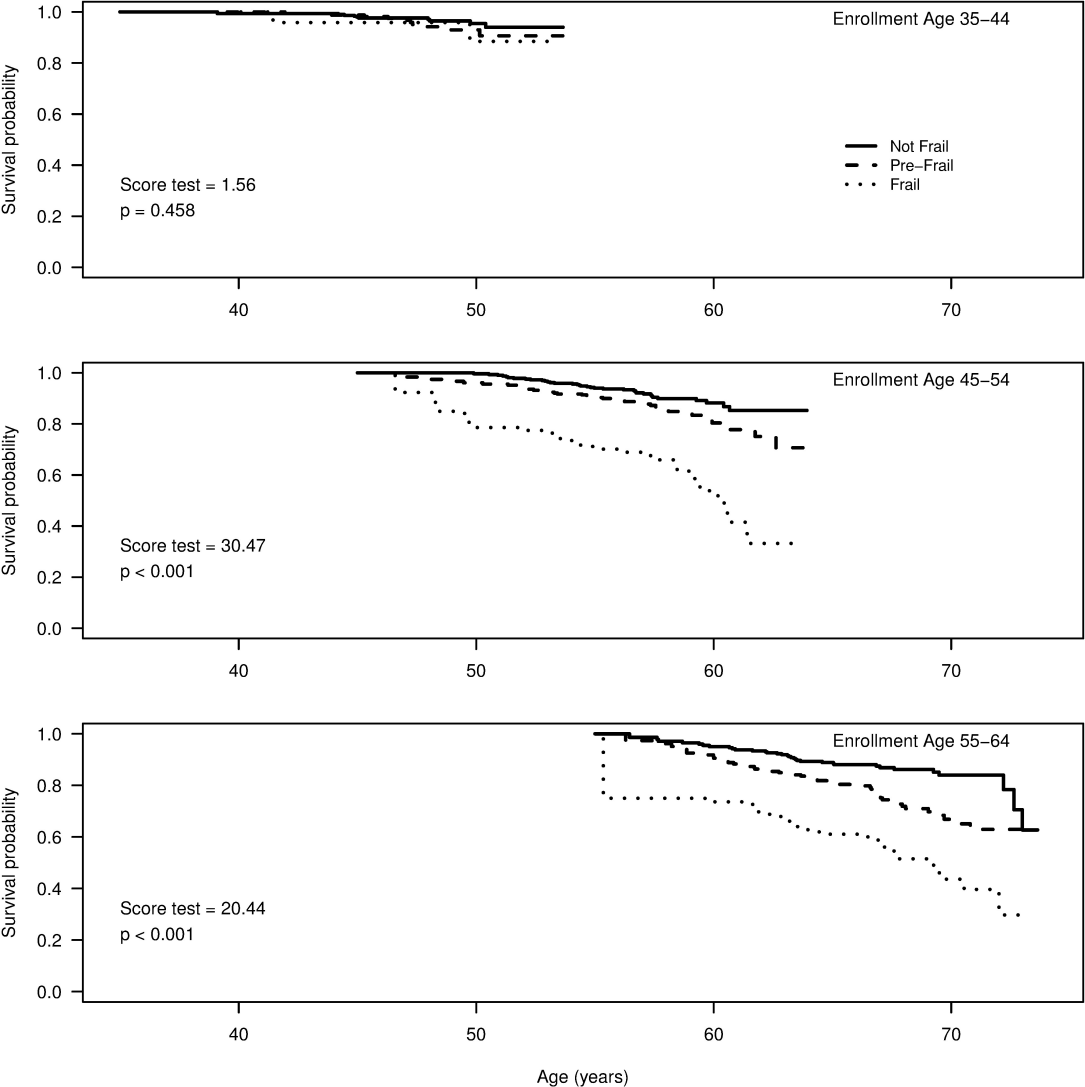
Overall survival by initial frailty group, stratified by age at enrollment.

Initial frailty was associated with survival (median 6.6 years) for the 2499 participants with follow-up data. Deaths were recorded for 55/271 (20%) frail participants, 97/908 (11%) pre-frail participants, and 78/1320 (6%) who were not frail. Crude mortality rates were 9 per 1000PY for not frail, 16 per 1000PY for pre-frail and 32 per 1000PY for frail participants. There were differences in overall survival probability associated with frailty group (Score test = 43.88, p<0.001) with the lowest survival probability for those in the frail group, intermediate survival probability for those in the pre-frail group, and the greatest survival probability for those who were not frail. Stratified analyses showed the differences among the frailty groups were evident in those initially aged 55–64 years (Fig 1; Score test = 20.44, p<0.001), and in those initially 45–54 years old (Score test = 30.47, p<0.001).

We also examined the association of frailty status (frail/not) with overall survival (Table 4). Frailty status alone was significantly associated with lower survival over the study period (HR = 2.36; 95%CI 1.74–3.20). The Cox proportional hazards model examining the relationship of frailty on survival along with sex, race, poverty status, BMI and educational attainment resulted in a final model with frailty, BMI and a three-way interaction of sex, race and poverty status (Table 4). Frailty had a similar association with survival in the adjusted model (HR = 2.30; 95%CI 1.67–3.18) as the unadjusted model, suggesting an independent association with survival beyond the influence of the other variables. Frailty status did not significantly interact with any of the other variables in the model. Survival was also associated with a three-way interaction of sex, poverty status and race as found in a previous HANDLS study [19]. Poverty status had a significant influence on survival only for African American men, with those living below poverty at baseline having more than twice the risk of mortality as those living above poverty (HR = 2.32; 95%CI 1.41–3.81). Having at least a high school education, or a BMI value above normal (overweight, obese class I), but below obese class II, was associated with a decreased hazard for mortality compared with normal BMI. No violations of the assumptions for proportional hazards were apparent in the full model by examining Schoenfeld residuals and this model met the proportionality assumption [20].

**Table 4:** All-cause mortality hazard ratios and 95% confidence intervals by frailty status and demographics.

	Hazard ratio	Lower 95% CI	Upper 95% CI
**Unadjusted**			
Frailty Status^1^ (frail)	2.36*	1.74	3.20
**Adjusted**			
Frailty Status (frail)	2.30*	1.67	3.18
Poverty status (below)	1.61	0.84	3.10
Sex (men)	2.16*	1.20	3.89
Race (African American)	1.13	0.60	2.12
BMI classes^2^			
Underweight	1.38	0.73	2.61
Overweight	0.50*	0.35	0.72
Class 1	0.42*	0.27	0.65
Class 2	0.71	0.49	1.04
HS graduate^3^	0.76*	0.58	0.99
Poverty: Race	0.95	0.41	2.19
Sex: Poverty	0.39	0.14	1.07
Sex: Race	0.64	0.28	1.46
Sex: Poverty: Race	3.92*	1.13	13.54

*p < .05

^1^Frail versus not or pre-frail

^2^Body mass index: Underweight BMI<18.5; Normal 18.5≤BMI<25 (reference group); Overweight 25≤BMI<30; Obesity class 1 30≤BMI<35; Obesity class 2 BMI≥35

^3^High-school graduate or obtained GED

## Discussion

This study examined the relationship of race and poverty status on frailty prevalence, and their influence on the association of frailty on survival. In the racially and economically diverse HANDLS cohort, frailty prevalence was higher for those living below poverty status. Race was only related to frailty prevalence for the younger participants, and in an unexpected direction with white participants being more likely to be frail than their African American counterparts. The association of frailty and survival was independent of the complex influence of sex, race and poverty status in this middle-aged cohort.

Frailty was more common among women, those with lower education, living below poverty, and among those taking multiple prescription medications. These findings are supported by previous work, usually with older people, indicating that frailty as assessed by the FRAIL scale is more common among women, lower education, lower income [6] and is associated with polypharmacy [17]. Even in this middle-aged cohort, the prevalence of frail individuals was higher in older age categories indicating increased susceptibility with age concordant with the literature [9, 21]. To our knowledge, this is the first study to examine the association of race and poverty with the FRAIL scale. Living below poverty more than doubled the odds of frailty for all participants, while race was only significant for those under age 55 with white participants having higher odds of frailty than African Americans. Our finding of greater frailty in younger white participants after adjusting for poverty status is novel. Although the novelty may be due to some peculiar regional attribute of our sample, it is more likely that this finding is unfamiliar because other studies have not examined frailty using balanced designs of age, sex, race, and poverty status. Previous work examining race and socioeconomic status with the Fried phenotype [9] found that African Americans had higher rates of frailty than whites for Cardiovascular Health Study (CHS) participants aged 65–74 years [9, 12], and for women in the Women’s Health and Aging Studies (WHAS) aged 70–79 [2]. Both of these studies, and others [22], examined people at older ages. The CHS cohort has a small percentage of African Americans (14%), and nearly two-thirds of their frail African Americans had low annual incomes (<$12,000). Thus, it is unclear how much income or poverty could account for the apparent racial differences in that cohort [12]. The WHAS sample had a larger percentage of African Americans (24%) and found that race was not significantly associated with frailty when any measure of socioeconomic status was included in the model. Frailty has been shown to be higher among those with low incomes regardless of race both in this study and others [2, 9, 22]. The trajectory of frailty prevalence over age may differ by race. This study demonstrated an increased prevalence of frailty for white participants compared with African Americans during middle-age. Studies in other diverse cohorts are necessary to validate this finding, and future studies in HANDLS can examine this trend over time.

HANDLS participants as young as 35–44 years old exhibited frailty; approximately 7% were categorized as frail in this community-dwelling cohort. The FRAIL scale has rarely been applied to middle-aged adults and this study provides an important extension of previous research. A study of Canadian adults found that 5.7% of those aged 35–49 years were frail according to the Fried criteria [23], and a study of Australian women identified 5.8% of those aged 50 years as frail [8]. Together these studies demonstrate a propensity for frailty before patients become elderly, and before medical professionals consider frailty as part of regular assessments [24]. The oldest participants in this study, aged 55–64 years, are younger than often considered for frailty assessment although 15% presented as frail. Identification is necessary for intervention which may slow or reverse the effects of frailty [25], especially for pre-frail individuals [26, 27].

Initial frailty groups (not, pre-frail, frail) were associated with survival over an average of 6.6 years, with frail individuals having the lowest survival, followed by pre-frail and then not frail. This is consistent with African American [6] (49–65 years old) and European [10] (40–79 years old) cohorts which have demonstrated this association in the FRAIL scale. The crude mortality rate in the HANDLS cohort for frail individuals was 3.5 times that for robust individuals, and twice the rate for pre-frail. Cox proportional hazards models demonstrated an increased risk for overall mortality for frail HANDLS participants, either alone or with demographic covariates. Frailty, defined as the FRAIL scale [6, 28], frail phenotype [9], or a frailty index [29], has been associated with an increased risk for mortality even for individuals who are not elderly [30]. This study expands the previous research by integrating race and poverty, known mortality risk factors [31, 32] which are also related to frailty. Frailty did not significantly interact with sex, race or poverty in accounting for mortality risk, indicating it has separate associations with survival beyond those explained by racial and economic factors.

Individuals in the overweight and class I obesity categories had a lower risk of mortality when frailty was in the model. This is another example of the obesity paradox defined as the observed survival advantage for obese individuals. Frail overweight and obese women in the Study Osteoporotic Fractures after 9 years of follow-up had lower hazard rates for mortality than frail women who were normal weight [33]. Other studies have shown that when lean tissue or muscle mass is taken into account the obesity paradox is eliminated [34].

Our findings are consistent with the notion of intrinsic capacity, the combination of individual physical and psychological capacities over time [1]. From this perspective, healthy aging is regarded from a functional viewpoint in which longitudinal trajectories of multifaceted traits are important assessments of health even before they exceed clinical thresholds. Intrinsic capacity views healthy aging from the perspective of functional abilities, the decline in which may lead to diagnosable diseases, but for which early treatment may present the transition to treatable conditions. Our data support the concept of intrinsic capacity by demonstrating that both pre-frailty and frailty are part of a continuum of physical incapacities which may emerge before old age. Our findings also extend this notion by showing that intrinsic capacity may vary by sex, race, and poverty status. Consequently, environmental influences on intrinsic capacity–including societal affects–provide opportunities for modifying functional trajectories to delay age-related decline.

This study has several limitations. The FRAIL scale is but one measure of the frailty concept. It was chosen for applicability to community health centers [13]. Comparison studies [10, 29] have shown great similarities in predicting mortality across a frailty index, Fried’s frailty phenotype [9], and the FRAIL scale [4]. HANDLS data on illnesses, functional status and physical abilities are self-reported and possibly under-reported. We were also limited by missing data, or participants who enrolled but then did not complete a full exam. The FRAIL components were similar to that used by Morley et al. [6] except for the weight loss component. Even with this change the FRAIL scale demonstrated construct validity with physical functioning, IADLs, grip strength and self-rated health, similar to previous validations [6, 35, 36]. The HANDLS cohort is from one location and may not be representative of other populations, although demographic comparisons suggest that the HANDLS cohort is representative of other US cities resembling Baltimore’s size and racial composition [37]. Nevertheless, HANDLS is a well-balanced bi-racial and economically diverse cohort enabling investigation into the independent effects of demographic variables which are highly correlated or confounded in other studies.

Our data support the notion that frailty is not just a geriatric syndrome but a continuum of signs and symptoms that emerge as early or perhaps earlier than mid-life. The previously recognized higher prevalence of frailty in African Americans may only hold for older ages, or be moderated by economic conditions. The FRAIL scale was significantly associated with survival in this cohort of older adults, independent of sex, race and poverty status. Recognition of frailty and pre-frailty before the traditional geriatric age may prevent disability and extend life expectancy in vulnerable groups.
